# General and Specific Contributions of RAN to Reading and Arithmetic Fluency in First Graders: A Longitudinal Latent Variable Approach

**DOI:** 10.3389/fpsyg.2017.01746

**Published:** 2017-10-06

**Authors:** Caroline Hornung, Romain Martin, Michel Fayol

**Affiliations:** ^1^Luxembourg Center for Educational Testing, Faculty of Language and Literature, Humanities, Arts and Education, University of Luxembourg, Esch-sur-Alzette, Luxembourg; ^2^UMR 6024, Laboratoire de Psychologie Sociale et Cognitive, Centre National de la Recherche Scientifique (CNRS), Clermont-Ferrand, France; ^3^University of Auvergne, Clermont-Ferrand, France

**Keywords:** rapid automatized naming, vowel RAN, finger-numeral configuration RAN, reading skills, arithmetic fluency, longitudinal study, structural equation modeling

## Abstract

In the present study, we opted for a longitudinal design and examined rapid automatized naming (RAN) performance from two perspectives. In a first step, we examined the structure of RAN performance from a general cognitive perspective. We investigated whether rapid naming measures (e.g., digit RAN and color RAN) reflect a mainly domain-general factor or domain-specific factors. In a second step, we examined how the best fitting RAN model was related to reading and arithmetic outcomes, assessed several months later. Finally in a third step we took a clinical perspective and investigated specific contributions of RAN measures to reading and arithmetic outcomes. While RAN has emerged as a promising predictor of reading, the relationship between RAN and arithmetic has been less examined in the past. Hundred and twenty-two first graders completed seven RAN tasks, each comprising visually familiar stimuli such as digits, vowels, consonants, dice, finger-numeral configurations, objects, and colors. Four months later the same children completed a range of reading and arithmetic tasks. From a general descriptive perspective, structural equation modeling supports a one-dimensional RAN factor in 6- to -7-year-old children. However, from a clinical perspective, our findings emphasize the specific contributions of RANs. Interestingly, alphanumeric RANs (i.e., vowel RAN) were most promising when predicting reading skills and number-specific RANs (i.e., finger-numeral configuration RAN) were most promising when predicting arithmetic fluency. The implications for clinical and educational practices will be discussed.

## Introduction

Rapid automatized naming is the ability to name a sequence of highly familiar visual stimuli such as colors, letters, and digits as fast as possible (Denckla and Rudel, 1974). Most of the existing studies on RAN focused either on its relation with academic outcomes, such as reading ability (Araújo et al., 2015, for a meta-analysis; Kirby et al., 2010; Norton and Wolf, 2012, for a review; Papadopoulos et al., 2016; for a review, Siddaiah and Padakannaya, 2015) and mathematics, especially arithmetic fluency (Hecht et al., 2001; Koponen et al., 2013, 2017; Foster et al., 2015; Cui et al., 2017) or on its underlying domain-general components (e.g., articulation time and pause time, Neuhaus et al., 2001a; Araújo et al., 2011). A remaining question is whether a range of different RAN tasks is best represented by a single component or/and by several specific components. The present paper contributes to determine both, the common part of all RANs and specific contributions related to different academic performances. One major goal is to provide practitioners criteria to choose among the variety of RANs (e.g., digits, colors, and letters) to better screen and predict children’s potential to success in reading and arithmetic.

### RAN, Reading, and Arithmetic

RAN and reading involve closely-related cognitive processes, such as attention to the stimuli, visual processes for recognition and discrimination, integration of visual information with stored orthographic and phonological codes, access and retrieval of phonological codes, articulation and serial processing (Manis et al., 1999; Wolf and Bowers, 1999; Norton and Wolf, 2012; Georgiou et al., 2013a). These common underlying processes appear to turn RAN into a promising predictor of early reading skills (de Jong and van der Leij, 1999; Manis et al., 1999; Kirby et al., 2003; Landerl and Wimmer, 2008; Lervåg and Hulme, 2009; Hornung et al., 2017) and reading development in both alphabetic and non-alphabetic writing systems (Siddaiah and Padakannaya, 2015). Several researchers emphasize that RAN is a unique predictor for reading (McBride-Chang and Manis, 1996) because RAN explains unique variance in reading after accounting for phonological awareness and letter knowledge (Scarborough, 1998; Kirby et al., 2003). Denckla and Cutting (1999) showed that poor readers could be sub-typed in those with phonological deficits only, with RAN deficits only, and in those who have both deficits. Accordingly, de Jong and van der Leij (2003) suggest that RAN is a powerful tool for identifying children at risk for reading difficulties (also see Bowers and Wolf, 1993; McBride-Chang and Manis, 1996; Shih et al., 2010; Norton and Wolf, 2012; Pan et al., 2013). Stainthrop et al. (2013) found that children who were performing lower on spelling tasks also performed lower on RAN tasks. Especially for clinical diagnosis and educational practices it is essential to identify and include the most predictive rapid naming measures into early readers’ screening batteries to support early identification and remediation of children at risk for developing reading difficulties.

The relationship between RAN and arithmetic has been less investigated in the past and research results have led sometimes to different conclusions. Koponen et al. (2017) concluded that the reason why RAN predicts arithmetic is that both share underlying processes, such as fast access to and retrieval of phonological representations stored in long-term memory. Some authors found that slower RAN was related to arithmetic difficulties (Van der Sluis et al., 2004) while others did not report this finding (Landerl et al., 2009). More recently, however, researchers report consistent associations between RAN and arithmetic fluency (Koponen et al., 2006, 2007, 2017).

Previous studies usually distinguished between non-alphanumeric and alphanumeric RAN when investigating their relation to reading and/or to arithmetic. Alphanumeric RAN refers to the rapid naming of familiar written symbols, such as letters and digits, usually faster named than non-alphanumeric stimuli (Denckla and Rudel, 1974). Non-alphanumeric rapid naming refers to the rapid naming of visual stimuli that are not written symbols, such as colors and objects. Researchers repeatedly observed stronger connections between alphanumeric RAN and reading outcomes than between non-alphanumeric RAN and reading outcomes (for a meta-analysis, see Meyer et al., 1998; Bowey, 2005; Araújo et al., 2015). Interestingly, Donker et al. (2016) explored whether RAN is a possible candidate to explain the overlap between reading and/or spelling, and mathematical difficulties in 133 primary school children. They reported that 7- to -10-year-old children, who were diagnosed with arithmetic difficulties, were only impaired on non-alphanumeric RAN, whereas children with reading and/or spelling difficulties and comorbidity were significantly slower on both types of RAN measures (i.e., non-alphanumeric and alphanumeric RAN). The authors thus highlight the importance to consider alphanumeric and non-alphanumeric RAN separately for diagnoses of individual disabilities and comorbid disabilities. The use of one general RAN factor may distort the specific problems children with learning difficulties have. A more detailed RAN profile therefore gives more comprehensive information on the cognitive impairments involved. In the same vein, Norton and Wolf (2012) underline that the rapid naming of colors and objects is less strongly correlated with reading performance than the rapid naming of letters and digits. One explanation is that the rapid naming of letters and digits is very similar to decoding and reading. Print-to-sound translations become increasingly faster when children are more exposed to letters and digits during formal schooling. This development might explain why reading fluency is more strongly related to alphanumeric RAN than to non-alphanumeric RAN (Norton and Wolf, 2012). Besides, colors and objects are usually not processed in a serial manner and thus the serial naming of colors and objects appears less automatized and less similar to reading. Furthermore, color and object naming require additional processes such as establishing meaning and selecting the correct name code before pronouncing a response (cf. Roelofs, 2006). However, prior studies did not show that arithmetic performance is stronger associated with alphanumeric RAN than with non-alphanumeric RAN. In line with Koponen et al. (2016), we suggest that non-alphanumeric RAN measures can be used as reliable early predictors of arithmetic fluency. It is possible, that children with arithmetic difficulties may have more difficulties in retrieving semantic information from memory than in transcoding visually presented symbols into phonological codes such as Arabic digits into spoken numbers (Donker et al., 2016; also see Slot et al., 2016). In line with this finding, Georgiou et al. (2013b) suggest that RAN is a marker of speed of access to representations in long-term memory rather than a marker of the ability to integrate information across different codes. By contrast, children with reading difficulties may have more difficulties to access print-to-sound translations and thus, have impaired alphanumeric RAN performances.

Few studies investigated the relationship between RAN, reading and arithmetic skills at the same time (e.g., Georgiou et al., 2013b; Koponen et al., 2016). Recently, Koponen et al. (2016) showed that kindergarteners’ verbal counting and RAN were unique and potential predictors of reading and arithmetic fluency 3 years later. The authors controlled for phonological awareness, receptive vocabulary, working memory, and socio-economic background. Relatedly, Georgiou et al. (2013b) found that kindergarteners’ RAN was a unique predictor for reading fluency but not for calculation fluency at the end of first grade when controlling for general cognitive ability, phonological awareness, processing speed, and working memory. Processing speed alone or in combination with phonological awareness and verbal short-term memory was sufficient to explain the RAN – math relationship. Accordingly, Georgiou et al. (2013b) suggest that RAN measures can be used as proxy measures of processing speed but that there is nothing in RAN that is exclusively related to mathematics. But they also note that the use of non-alphanumeric RAN (colors and objects) may have inflated its relationship with processing speed, because object RAN involves more general processing speed compared to letter RAN (Neuhaus et al., 2001b). In both above-mentioned studies, RAN performance was assessed in children before formal alphabetization only by non-alphanumeric RAN tasks (e.g., color RAN) to study their later contribution to reading and arithmetic. Usually, researchers administer object and color RAN in children before formal education, because at that age, children do not yet read and only start learning the numbers and the alphabet. Consequently the associations between RAN, reading and arithmetic in children crucially depend on the RAN and outcome measures used in the study and on the children’s age. It is therefore possible, that the additional use of alphanumeric RAN may lead to different associations between RAN, reading and arithmetic.

Rapid naming in children has been generally measured by the four standard RAN measures, naming colors, objects, digits or letters. As mentioned before, kindergarten children are still learning the numbers and the alphabet, and they frequently perform color and object RAN tasks (e.g., Georgiou et al., 2013b) while primary school children usually perform letter and digit RAN tasks. Recently, Hornung et al. (2017) reported that vowel RAN assessed in kindergarten and first grade, was a stronger predictor for reading in first and second grade, than consonant RAN. Vowels are easier to produce and children are more familiar with vowels than with consonants at the beginning of formal education. We therefore suggest that in beginning readers vowel rapid naming is probably more informative than a combined letter RAN. We therefore suggest that the child’s familiarity with the stimuli but also of the nature of visually presented stimuli (symbolic-alphanumeric, symbolic-non-alphanumeric, and non-alphanumeric) are crucial for RAN performance and its relation to reading and arithmetic.

Arithmetic has been similarly related to alphanumeric and non-alphanumeric RAN (e.g., Koponen et al., 2013). Nevertheless, it is possible that RAN tasks including number-specific stimuli such as for example dice naming or finger-numeral configuration naming, already familiar to young children, might more strongly predict arithmetic performance than letter, digit, color or object naming (also see, Cui et al., 2017). To perform well on these number-specific naming tasks, children have to quickly process the quantities from 1 to 5 of these configurations, access, and retrieve their respective names form long-term memory. Subitizing and counting small sets of items (e.g., dots and fingers) are indices of core number competences underlying later math development (Feigenson et al., 2004; Gray and Reeve, 2014; Hornung et al., 2014; Penner-Wilger et al., 2014). For example, Gray and Reeve (2014) found that preschoolers with weak subitizing profiles showed poorer arithmetic ability. Additionally, finger representations have been associated with the development of exact number processing (Fayol et al., 1998; Noël, 2005; Krinzinger et al., 2011). Likewise, Di Luca and Pesenti (2008) suggest that canonical finger-numeral configurations may be linked to exact number magnitudes during cognitive development and finally turn into abstract symbols (also see Di Luca and Pesenti, 2011). From this view, a weak enumeration and subitizing profile for dots and fingers may have diagnostic significance in preschoolers to identify later arithmetic difficulties. When compared with typically developing children, children with arithmetic difficulties might require significantly more time to retrieve semantic information from memory (i.e., a quantity) in order to name dice and finger-numeral configurations than to transcode Arabic digits into spoken numbers (cf. Schleifer and Landerl, 2011; Slot et al., 2016). Thus, adding quantitative-numerical RAN measures such as dice RAN and finger-numeral configuration RAN to the usually administered RAN measures, may further enlighten the RAN – arithmetic relationship in typically developing children at the beginning of formal education.

### The Present Study

To date, no studies have explored the latent structure of RAN and whether one or several RAN factors are needed to predict academic outcomes, such as reading and arithmetic. Investigating this question at the beginning of formal schooling when children start to learn to read and to calculate, is particularly interesting with respect to early screening and educational practices, which aim to identify children at risk at an early stage of developing reading and/or arithmetic difficulties. From a general cognitive perspective, the present study investigates firstly whether the performance collected on a large set of RANs (i.e., colors, objects, dice patterns, canonical finger-numeral configurations, vowels, consonants, and digits) is mainly underpinned by a domain-general factor or multiple related factors. We will therefore examine the structure of RAN investigating five different possible latent models.

In the following, we briefly describe these five models. Model 1 examines the hypothesis of a unique RAN component to account for the variance of all seven administered RAN measures with different task stimuli. A single RAN factor model represents the most parsimonious model to explain the cognitive processes involved in first graders’ rapid automatized naming. Model 2 examines the repeatedly reported distinction between non-alphanumeric RAN (color, object, and dice finger-numeral configuration) and alphanumeric RAN (digit, vowel, and consonant) measures (Norton and Wolf, 2012). Model 3 examines the distinction between non-symbolic RAN (color and object) and symbolic RAN (digit, vowel, consonant, dice, and finger-numeral configuration) measures. No studies to date investigated RAN from this view. We hypothesize that the model fit will be adequate because symbolic RAN is assessed by stimuli that are processed as symbolic codes (e.g., digits, dice pattern, canonical finger pattern, and vowels) frequently used by first graders. Model 4 examines the distinction between non-symbolic non-alphanumeric RAN (color and object), symbolic non-alphanumeric RAN (dice and finger-numeral configuration), and alphanumeric RAN (digit, vowel, and consonant) measures. No studies to date investigated RAN from this view. However, prior findings distinguished between alphanumeric and non-alphanumeric RAN. Here we add a third factor assessed by symbolic number-specific RAN involving numerosity naming. Model 5 examines the distinction between letter RAN (vowel and consonant) and non-letter RAN (digit, dice, finger-numeral configuration, color, and object). Prior findings reported stronger correlations between reading and letter RAN than between reading and other RAN measures such as digit or object RAN (de Jong, 2011; Hornung et al., 2017; but see Vaessen and Blomert, 2013). Thus, we would like to test whether letter RAN can be distinguished from non-letter RAN.

Secondly and complementary, we will draw on the best fitting RAN model from the previous analysis to study the latent relationships between RAN, reading accuracy, reading speed, and arithmetic fluency in first graders. To the best of our knowledge, the structure of RAN and the latent relationships between RAN, reading, and arithmetic have not yet been investigated to date in beginning readers. Finally, in a last step we will opt for a predictive “clinical” approach, to determine whether certain RANs are sounder measures to predict reading and arithmetic skills at the beginning of formal instruction. To investigate this question, we will conduct a path analysis on all observed variables to evaluate the relationships between RAN measures and academic outcome variables.

## Materials and Methods

### Participants

One hundred twenty-two first graders (60 girls and 62 boys) from a mainstream school located in the XX district in Paris participated in the present study. Age ranged from 68 to 86 months (*M* = 75,73 months; *SD* = 3,74 months). All children came from middle socio-economic backgrounds (based on the location of schools) and were native French speaking children. Teacher interviews informed that the participating children had neither neurological, psychiatric or behavioral problems, nor school delays. The present study has been carried out in accordance with appropriate ethical principles and standards. The Ethics Review Panel of the University of Pierre et Marie Curie approved the study and in accordance with the Ethics Review Panel, we obtained written consent from all the parents and verbal consent from all the children and teachers prior to task administration.

### Measures

We selected tasks appropriate and comprehensive for first graders based on prior studies and discussions with elementary school teachers. Forty children performed all tasks, except the “read the words” task twice as to establish a measure of test-retest reliability.

#### Rapid Automatized Naming

In total seven different RAN measures were administered. The test material was developed on the basis of Denckla and Rudel (1974). We used the standard color, object, and digit rapid naming measures. Additionally, we divided the letter RAN in a vowel and a consonant RAN because beginning readers have more facility in naming vowels than consonants, and thus yielding stronger associations between RAN and reading (Hornung et al., 2017). Furthermore, we added two measures involving number-specific material, finger-numeral configurations, and dice patterns. Children had to name as fast as possible five recurring colors (red, blue, green, black, and yellow), objects (dog, foot, tree, book, and table), vowels (A, E, I, O, and U), consonants (C, L K, P, and R), digits (1–5); finger-numeral configurations (1–5; see **Appendix 1**), and dice configurations (classic patterns 1–5). For all the RAN tasks, the stimuli were randomly arrayed in five rows of eight on a separate sheet, for a total of 40 items. All tasks were paper-pencil. RTs were recorded via electronic chronometer. RAN tasks were used in their serial administration procedure because they are usually administered serially when investigating their relationship with reading or to identify children at risk for reading difficulties. When stimuli are presented individually, the RAN performance means something different than the performance we wanted to measure: that is serial and fast familiar stimuli naming as you do when reading a text or an arithmetic problem. Cronbach’s Alpha was 0.90 for the seven RAN measures. Before recording the children’s reaction times on the RAN task, children were asked to practice and to name the five possible stimuli on a separate practice sheet to ensure familiarity. After practice, reaction times were recorded on each RAN measure. The number of errors was recorded but overall small and not further considered in the analyses. That is to say, mean error rates were lowest in dice RAN (*M* = 0.18; *SD* = 0.51), digit RAN (*M* = 0.19; *SD* = 0.59), finger RAN (*M* = 0.19; *SD* = 0.56) and color RAN (*M* = 0.24; *SD* = 0.63), and highest in vowel RAN (*M* = 0.98, *SD* = 3.64) and consonant RAN (*M* = 4.14; *SD* = 7.32). Test-retest reliability coefficients of the RAN measures were all above 0.75.

#### Reading Outcomes

In the *1-min task* (Khomsi, 1999) children had 60 s to read 55 pseudowords. Thirty-one pseudowords were composed of one syllable (e.g., cal) and 24 pseudowords of two syllables (e.g., ousir). The score was the total amount of correctly read “words” in 60 s. Test-retest reliability was 0.80.

In the *read the words* task the children had to read six rows, each comprising five words (e.g., row 1: “porte, table, minute, samedi, livre, arbre”; in English: “door, table, minute, saturday, book, tree”). After each row, reading accuracy and reading time were recorded. The resulting six accuracy and speed scores were used to establish the Cronbach’s alpha (α = 0.87) and were then averaged afterward as to have one measure for reading accuracy and speed, respectively.

In the *read the text* task the children had to read a brief text of three sentences (23 words) (e.g., “Gaston le cochon achète une voiture rouge. Tout content, il remplit un panier de salades. Gaston va voir son ami Louis le lapin.” In English: “Gaston the pig buys a red car. Happy, he fills a bucket with salads. Gaston goes to see his friend Louis the rabbit”). Text reading time for the three sentences was recorded for each child, resulting in a text reading time score. We also recorded errors on the *read the text* task to compute a text reading accuracy measure. Test-retest score was 0.75.

#### Arithmetic Outcomes

Arithmetic fluency was measured by 33 additions and 33 subtractions, visually presented in 3 columns on 2 separate sheets (font: Arial; font size: 14; color: black). Children had to write the response behind each arithmetic problem. Item difficulty progressively increased per column. For example in the first column, additions were basic problems up to 10 (e.g., 4 + 6 = ?), in the second column, additions were problems up to 20 (9 + 7 = ?) and in the third column, additions were problems up to 100 (e.g., 55 + 24 = ?). Subtraction problems were developed in a similar way with problems of progressive difficulty per column. Children first had to solve and write down as many additions correctly as possible in 90 s and then to solve as many subtractions correctly as possible in 90 s. Both arithmetic tasks were each preceded by two easy practice trials (e.g., 2 + 2 = ?; 3 - 1 = ?). The score was the total amount of correctly solved calculations. The maximum possible score was 33 points per task. Test-retest score was 0.85 for the addition task and 0.90 for the subtraction task.

#### Processing Speed

To measure general processing speed children performed a shape comparison task on a separate sheet of paper before performing the arithmetic tasks (cf. letter comparison task in adults, Cepeda et al., 2013). Several training items preceded the tasks. Children compared pairs of two visually presented shapes (e.g., square, heart, triangle, and circle). They wrote an equal sign ( = ) between both shapes when these were the same, but a slash sign (/) when both shapes were different. The children had 30 s to compare as many pairs of shapes as possible (maximum = 48). The score was the total number of correct answers. Test-retest score was 0.90.

### Procedure

The children completed all seven RAN tasks, processing speed, reading and arithmetic tasks in their schools. The test administration was divided in three distinct sessions. Test session 1 was an individual session and took place in a quiet room at the school in October. In the first session the children performed seven different RAN tasks. To prevent a possible bias in administration order, we controlled the order of presentation of the RANs by rotating the successive RAN tasks. There were seven possible administration orders. One administration order was for example the naming of colors, objects, vowels, consonants, digits, finger configurations, and dice patterns whereas the next administration order started with the naming of objects and ended with the naming of colors, and so on. Test sessions 2 and 3 took place 4 months later. Test session 2 was a group session assessing children’s processing speed and arithmetic performance. It took approximately 10 min. In test session 3 children were individually evaluated on their reading abilities. Both individual test sessions lasted approximately 20 min per child. Each task started with several practice examples and the test did not start until the child fully understood the task instructions. Children were not given any performance-contingent feedback during the administration. However, the experimenter gave general support independent of the child’s performance.

### Statistical Analyses

Structural equation modeling (SEM) is a promising statistical method because it reduces measurement error and allows estimating the fit of theoretical models. According to Bollen (1989), the variance common to a set of related measures gives a more coherent representation of an underlying theoretical construct than any single measure of a task as task-specific variance is not represented in the variance of the latent construct. Moreover, latent variables are free of measurement error. That is to say, relative to traditional regression analyses, SEM provides an unbiased estimate of the longitudinal relationships between a predictor and outcomes variables (cf. Cohen et al., 2003). Given these advantages, in the following we run a series of different structural equation models to study the structure of RAN and its association with reading and arithmetic outcomes 4 months later. All structural equation models were estimated with the software Mplus 5.2 (Muthén and Muthén, 1998–2007). Model fit was evaluated by various indices: the chi-2 goodness-of fit statistic, the root-mean-square error of approximation (RMSEA), the comparative fit index (CFI), the Tucker-Lewis index (TLI), and the standardized root-mean-square residual (SRMR). A non-significant chi-2 goodness-of fit statistic indicates a good fit. The corresponding probability value indicates the probability of finding a multivariate difference of a certain size between the specified model and the sample data given that the specified model is the “true” model in the population. RMSEA values below 0.05 indicate a good model fit (Browne and Cudeck, 1993). CFI and TLI values larger than 0.95 and SRMR values close to 0.08 indicate a good fit (Hu and Bentler, 1999).

In order to investigate the respective contributions of RAN measures on first graders’ reading and arithmetic skills, we conducted a path analysis controlling for age and processing speed. Due to the longitudinal nature of the data collection, the criterion of time precedence was met, allowing us to specify the directionality of the presumed effects (as formulated by Kline, 1998). We then analyzed the path coefficients from all the independent variables to the dependent variables, resulting in a saturated model. The focus of this analysis is not the estimation of model fit, but the estimation of the contribution of the specific RANs and the variance in reading and arithmetic that can be explained (*R*^2^). The analysis of the path model was conducted using *MPlus* (Muthén and Muthén, 1998–2013). The seven RAN measures, age, and processing speed were entered as independent variables and the three academic outcomes (reading accuracy, speed, and arithmetic fluency) were entered as dependent variables. Only observed variables were entered into the analysis, no latent variables were included. Maximum likelihood estimation was performed.

## Results

The descriptive results and Pearson bivariate correlations for RAN and academic outcome measures are presented in **Table 1**. We controlled for age. All measures met standard criteria of univariate normality with skewness for all measures below 3 and kurtosis for all measures below 4 (Kline, 1998). We performed additional multicollinearity analysis on all 7 inter-correlated RAN measures (see Appendix 2). Variance inflation indices (VIF) provide a measure on how much the variance of an estimated regression coefficient is increased due to collinearity. The VIF indices here were all below 4 for all RAN measures, indicating that multicollinearity was not extreme and not a problem to perform regression analyses (O’Brien, 2007).

**Table 1:** Intercorrelations and descriptive statistics (*N* = 122 first graders).

Measures	1	2	3	4	5	6	7	8	9	10	11	12	13	14	15
**Rapid naming measures**															
(1) Color RAN	–														
(2) Object RAN	**0.66^∗∗^**	–													
(3) Vowel RAN	**0.69^∗∗^**	**0.60^∗∗^**	–												
(4) Consonant RAN	**0.56^∗∗^**	**0.53^∗∗^**	**0.64^∗∗^**	–											
(5) Digit RAN	**0.63^∗∗^**	**0.60^∗∗^**	**0.58^∗∗^**	**0.51^∗∗^**	–										
(6) Dice RAN	**0.69^∗∗^**	**0.68^∗∗^**	**0.73^∗∗^**	**0.54^∗∗^**	**0.71^∗∗^**	–									
(7) Finger-numeral RAN	**0.56^∗∗^**	**0.53^∗∗^**	**0.60^∗∗^**	**0.44^∗∗^**	**0.60^∗∗^**	**0.63^∗∗^**	–								
**Academic measures assessed 4 months later**											
**Reading accuracy**
(8) One-minute test	**-0.26^∗^**	**-0.29^∗^**	**-0.44^∗∗^**	**-0.38^∗∗^**	**-0.35^∗∗^**	**-0.31^∗∗^**	**-0.23^∗^**	–							
(9) Word reading accuracy	**-0.24^∗^**	**-0.31^∗^**	**-0.43^∗∗^**	**-0.48^∗∗^**	**-0.34^∗∗^**	**-0.28^∗^**	**-0.22^∗^**	**0.76^∗∗^**	–						
(10) Text reading accuracy	**-0.23^∗^**	**-0.33^∗^**	**-0.41^∗∗^**	**-0.42^∗∗^**	**-0.35^∗∗^**	**-0.28^∗^**	**-0.22^∗^**	**0.71^∗∗^**	**0.85^∗∗^**						
**Reading speed**															
(11) Word reading speed	**0.28^∗^**	**0.30^∗∗^**	**0.37^∗∗^**	**0.32^∗∗^**	**0.42^∗∗^**	**0.27^∗^**	0.16	**-0.73^∗∗^**	**-0.66^∗∗^**	**-0.65^∗∗^**	–				
(12) Text reading speed	**0.27^∗^**	**0.25^∗^**	**0.29^∗^**	**0.30^∗∗^**	**0.40^∗∗^**	**0.24^∗^**	0.09	**-0.71^∗∗^**	**-0.64^∗∗^**	**-0.62^∗∗^**	**0.89^∗∗^**	–			
**Arithmetic**															
(13) *Addition*	**-0.23^∗^**	**-0.25^∗^**	**-0.27^∗^**	**-0.23^∗^**	**-0.15^∗^**	**-0.26^∗∗^**	**-0.26^∗^**	**0.33^∗∗^**	**0.28^∗^**	**0.30^∗^**	**-0.29^∗∗^**	**-0.25^∗∗^**	–		
(14) *Subtraction*	-0.08	**-0.26^∗^**	**-0.22^∗^**	**-0.21^∗^**	-0.08	**-0.22^∗∗^**	**-0.29^∗^**	**0.39^∗∗^**	**0.38^∗∗^**	**35^∗∗^**	**-0.29^∗∗^**	**-0.25^∗∗^**	**0.48^∗∗^**	–	
(15) **Processing speed**	-0.07	-0.03	-0.09	-0.10	-0.09	-0.11	-0.16	-0.14	-0.12	-0.03	**0.21^∗^**	**0.26^∗^**	**0.28^∗^**	0.09	
*Mean*	51.52	58.59	47.74	58.23	40.72	43.42	46.77	17.70	3.47	15.74	30.87	84.86	11.23	7.80	14.84
*SD*	16.31	13.81	15.84	22.79	11.17	11.58	11.05	8.59	1.20	6.45	19.91	47.87	4.50	5.54	7.64
*Skewness*	1.21	0.40	1.20	1.37	1.01	0.94	0.93	0.67	-0.69	-1.13	1.65	1.12	-0.50	0.07	1.21
*Kurtosis*	1.11	-0.64	1.40	2.29	0.79	1.05	0.98	0.49	0.15	0.48	3.10	1.65	-0.02	-1.06	2.70


*Pearson correlations reaching significance at the 0.05 alpha levels are bold, ^∗∗^*p* < 0.001 and ^∗^*p* < 0.05. Age has been controlled for. RAN, rapid automatized naming.*

Several correlations are worth mentioning. All RAN measures are strongly and significantly intercorrelated ranging from *r* = 0.44 to *r* = 0.73, *p* < 0.001. Furthermore, all RAN measures are significantly related to reading accuracy (*r* = -0.22 to *r* = -0.48; *p* < 0.05) and speed (*r* = 0.24 to *r* = 0.42; *p* < 0.05), except finger-numeral RAN is not significantly correlated with reading speed. All RAN measures are also significantly related to arithmetic measures (*r* = -0.15 to *r* = -0.29; *p* < 0.05) except for color and digit RANs that did not significantly correlate with subtraction. All academic measures were significantly correlated |*r* = 0.25 to *r* = 0.89, *p* < 0.001|.

Digit RAN yielded the lowest mean reaction time on all RANs followed by dice RAN, and finger-numeral RAN. This result supports our hypotheses that dice and finger-numeral configurations are reliable measures for assessing naming speed with number-specific material in 6-year-old children.

### Structural Equation Modeling

Our SEM results are presented in two sections. In the first section, we present five different structural equation models investigating the structure of RAN in first grade children. In the second section, we extend the best fitting structural model of RAN and include reading outcomes (accuracy and speed) and arithmetic fluency to determine how RAN components relate to different academic performances.

#### The Structure of RAN

Generally, the five RAN models provided an excellent fit to the data, except Model 4 (see below). All model solutions were then “properly identified” as the estimation procedures converged, no parameter estimates were out of the range of admissible parameter estimates (e.g., negative variances, correlations greater than 1), and all matrices of parameter estimates were positive definite. Fit indices for all RAN models are shown in **Table 2**.

**Table 2:** Fit statistics of models capturing the structure of RAN controlled for age.

Model	*X*^2^	*df*	*p*	AIC	RMSEA	TLI	CFI	SRMR
Model 1 (RAN)	14.35	20	0.81	6324.94	0.00	1.00	1.00	0.02
Model 2 (RAN alphanumeric–RAN non-alphanumeric)	14.07	18	0.72	6328.66	0.00	1.00	1.00	0.02
Model 3 (RAN non-symbolic–RAN symbolic)	12.07	18	0.84	6326.66	0.00	1.00	1.00	0.02
Model 4 (RAN non-alphanumeric symbolic–RAN non-symbolic–RAN alphanumeric)	9.97	14	0.76	6332.56	0.00	1.00	1.00	0.02
Model 5 (RAN letters–RAN no letters)	12.70	18	0.81	6327.29	0.00	1.00	1.00	0.02
Cut-off criterion			>0.05		<0.05	>0.95	>0.95	<0.08


**X*^*2*^, chi-square goodness-of-fit statistic; *df*, degrees of freedom; AIC, Akaike; RMSEA, root-mean-square error of approximation; TLI, Tucker-Lewis index; CFI, comparative fit index; SRMR, standardized root-mean-square residual. A non-significant *X*^*2*^ statistic (*p* > 0.05) indicates a good fit of the model to the data.*

Model 1 investigated a unique and general RAN factor in children and provided an excellent fit to the data. This result is in line with the idea of a unitary RAN component. Statistically significant standardized factor loadings ranged from λ = 0.64 to λ = 0.88 for a unique RAN factor. Model 2 distinguished between non-alphanumeric RAN and alphanumeric RAN and provided an excellent fit to the data. Both factors were perfectly correlated (*r* = 1.00, *p* < 0.001). *Model 3* distinguished non-symbolic RAN and symbolic RAN and provided an excellent fit to the data. Both factors were also highly correlated (*r* = 0.96, *p* < 0.001). *Model 4* distinguished between non-symbolic RAN, symbolic non-alphanumeric RAN, and alphanumeric RAN measures. Symbolic non-alphanumeric RAN and alphanumeric RAN yielded a correlation greater than 1. Thus, Model 4 was not admissible. We introduced a correlation between the residual terms of the vowel and the consonant RAN because both involve letters. However, this modification did not solve the problem. We then allowed the vowel RAN and digit RAN residual variances to correlate because both measures were more strongly correlated than vowel and consonant RANs (cf. **Table 1**). This reduced the “greater than 1 correlation” and yielded a good fit to the data. One reason for this stronger vowel-digit RAN correlation is that children at this stage are more familiar with digits and vowels than with consonants. All three factors were highly correlated (*r* = 0.91 to *r* = 0.97). Model 5 investigated the domain-specificity of RAN by distinguishing between letter RAN and non-letter RAN. Model 5 yielded an excellent fit to the data, with a latent correlation of 0.95 between both factors.

**FIGURE 1:**
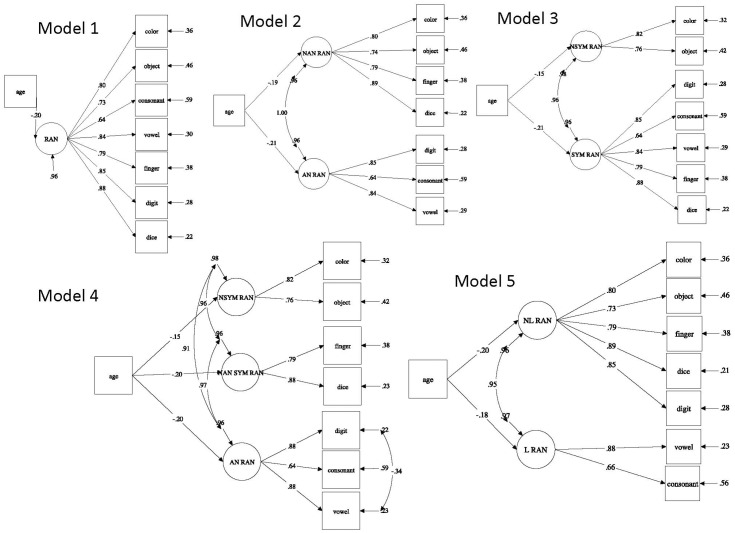
Five conceptually different structural equation models testing distinct RAN components in first graders. Latent variables RAN, rapid automatized naming; AN RAN, alphanumeric rapid automatized naming; NAN RAN, non-alphanumeric rapid automatized naming; NSYM RAN, non-symbolic rapid automatized naming; SYM RAN, symbolic rapid automatized naming; NAN SYM RAN, non-alphanumeric symbolic rapid automatized naming; NL RAN, non-letter rapid automatized naming; L RAN, letter rapid automatized naming.

Standardized parameter estimates for Models 1–5 are shown in **Figure 1**. Overall, our data suggests that all RAN components in Models 2–5 were highly correlated and appear to reflect the same underling processes. Fit indices for the five RAN Models were comparable. Thus, for reasons of parsimony we select Model 1 as the best fitting model for explaining general rapid naming in children.

#### The General Contribution of RAN to Reading and Arithmetic

**FIGURE 2:**
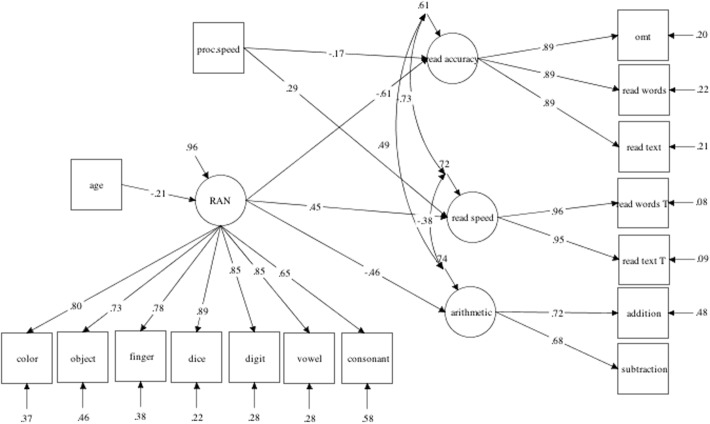
The best fitting structural equation model illustrating the contribution of RAN to reading accuracy, reading speed, and arithmetic. Only significant coefficients and relationships are depicted. proc. speed, processing speed.

The second purpose of the present study was to investigate how RAN would predict academic outcomes. We therefore chose Model 1, the best fitting and most parsimonious model with a single RAN factor. We extended this model and added the three academic outcome factors representing reading accuracy, reading speed and arithmetic, and controlled for age and processing speed. This latent longitudinal model with all predictor and outcome variables is presented in **Figure 2**. The fit statistics of this model were adequate (*x*^2^ = 129.06; *df* = 92, *p* = 0.001; RMSEA = 0.06; CFI = 0.97; TLI = 0.96; SRMR = 0.06). In this model 39% of the variance of reading accuracy, 28% of the variance in reading speed and 26% of the variance in arithmetic was explained. All factor loadings were significant. Age did not significantly influence the academic outcome variables although RAN significantly predicted all three outcomes and processing speed significantly predicted reading outcomes.

#### The Specific Contribution of RAN Measures to Reading and Arithmetic

The third goal of the present study was to investigate how different RAN measures would predict academic outcomes. We therefore conducted a path analysis with a series of regression analyses. Beforehand, we computed three mean composite scores for the academic outcome measures by averaging the three scores of reading accuracy, the two scores of reading speed, and the two scores of arithmetic performance to obtain one measure for reading accuracy, speed, and arithmetic fluency, respectively.

The seven RAN measures, age, and processing speed were entered as independent variables; the academic outcomes were entered as dependent variables.

**FIGURE 3:**
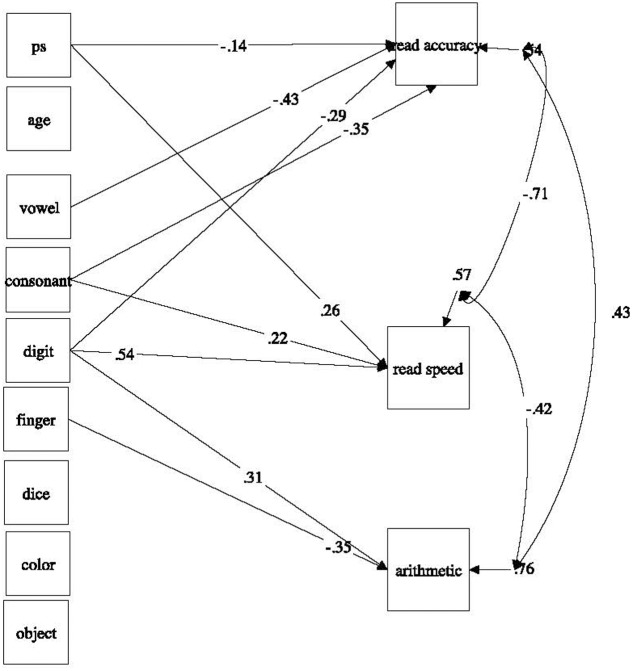
Specific RAN contributions to reading and arithmetic skills: A path model with significant standardized path coefficients. PS, processing speed.

Standardized path coefficients are presented in **Table 3**. The resulting path model showing only the significant paths and the corresponding standardized coefficients is presented in **Figure 3**. RAN and processing speed accounted for 46% of the variance in reading accuracy (*p* < 0.001), 43% of the variance in reading speed (*p* < 0.001), and 24% of the variance in arithmetic fluency (*p* < 0.01). Interestingly, processing speed predicts only reading outcomes significantly, but not arithmetic. All three alphanumeric RAN uniquely predict reading accuracy RAN measures (vowels, consonants, and digits). Reading speed is uniquely predicted by consonant and digit RAN but not vowel RAN. As hypothesized, arithmetic fluency is uniquely predicted by finger-numeral configuration and digit RAN, although dice RAN is not a unique predictor. In the following we will further discuss the main findings.

**Table 3:** Standardized path coefficients predicting performance in reading accuracy, reading speed, and arithmetic fluency.

	Reading accuracy	Reading speed	Arithmetic fluency
**Standardized path coefficients (standard error)**
Age	-0.03 (0.07)	-0.01 (0.07)	0.04 (0.08)
Processing speed	-0.014* (0.07)	0.26** (0.07)	0.12 (0.08)
Color RAN	0.21 (0.11)	-0.03 (0.12)	0.18 (0.13)
Object RAN	-0.01 (0.10)	-0.03 (0.11)	-0.19 (0.12)
Vowel RAN	-0.43** (0.12)	0.25 (0.13)	-0.08 (0.14)
Consonant RAN	-0.35** (0.09)	0.22* (0.11)	-0.20 (0.10)
Digit RAN	-0.29* (0.12)	0.54** (0.12)	0.31* (0.14)
Finger RAN	0.05 (0.11)	-0.22 (0.11)	-0.35* (0.12)
Dice RAN	0.12 (0.13)	-0.13 (0.14)	-0.13 (0.16)


**N* = 122. ^∗^*p* < 0.05; ^∗∗^*p* < 0.001, two-tailed.*

## Discussion

Our research objective was threefold. Firstly, we intended to determine whether seven different RAN tasks rely on one general RAN component or on different factors underpinning the performances of first graders. Secondly, we were interested in exploring the general latent relationships between RAN components and beginning reading accuracy (e.g., reading words and pseudowords), reading speed (e.g., single word and continuous text reading), and arithmetic fluency (e.g., addition and subtraction problem solving). Thirdly, we were interested in exploring the specific contributions of individual RAN measures on reading and arithmetic outcomes at the start of formal primary instruction.

As a general result, we found that all seven RAN performances drew on one unique factor. This unique factor was significantly predictive of reading and arithmetic outcomes. It reflects the shared processes from all seven RAN tasks which are for instance attention to the stimuli, visual processes for recognition and discrimination, integration of visual information with stored orthographic and phonological codes, access and retrieval of phonological codes, articulation and serial processing. By contrast, from a clinical perspective this “general” result is insufficient and does not give a clear indication of which RAN measure to choose when screening children at risk for learning difficulties. As the path analysis revealed, certain RAN measures are sounder predictors than others. Thus, choosing a RAN task randomly would not be the best method when aiming to identify children at risk for learning difficulties. Our findings emphasize that reading accuracy and reading speed were more strongly predicted by alphanumeric RANs when controlling for processing speed. Furthermore, arithmetic fluency was more strongly associated with number-specific RAN measures (i.e., finger-numeral configurations and digit RAN) when controlling for processing speed. As a consequence exploring the specificities of different RANs, leads to different and complementary conclusions whether RAN performance is observed from a general cognitive perspective or from a clinical diagnostic perspective.

Initially, inspired by prior research findings, we suggested that letter RANs are important predictors for reading ability, while digit or number-specific RANs might be promising specific predictors for arithmetic ability. The results show that general RAN is a promising and significant predictor for early reading and arithmetic skills when controlling for processing speed. However, the path analyses showed that only alphanumeric RANs significantly predicted reading outcomes when controlling for non-alphanumeric RANs and processing speed. Vowel RAN was the strongest predictor for reading accuracy (β = -0.43; *p* < 0.001), while digit RAN was the strongest predictor for reading speed (β = 0.54; *p* < 0.001). In relation to arithmetic fluency the path analysis emphasized the predictive value of finger-numeral configuration RAN (β = -0.35; *p* < 0.001) and digit RAN (β = 0.31; *p* < 0.001) in the beginning of formal schooling when controlling for processing speed and other RANs. This finding is in line with Koponen et al. (2013, 2017) showing that arithmetic performance involves conceptual and phonological processing and therefore similarly relates to alphanumeric and non-alphanumeric RAN. While, Koponen et al. (2017) showed that color and object RAN predicted arithmetic performance in kindergarteners, we found that finger-numeral configuration RAN was the soundest RAN measure to predict arithmetic in first graders when controlling for other non-alphanumeric and alphanumeric RANs.

By adding quantitative-numerical RAN measures such as dice RAN and finger-numeral configuration RAN to the generally administered RAN measures, we aimed to further explore the RAN – arithmetic relationship. Both tasks were significantly correlated with arithmetic measures. However, as mentioned above, finger-numeral configuration naming was a unique predictor for first graders arithmetic performance, while dice naming was not. Following work Di Luca and Pesenti’s (2011), finger-numeral representations are more than just another way to mentally represent quantities. They argue that finger-numeral representations contribute, if practiced at an early stage, to a fast and deep understanding of number concepts rooted in perceptual and sensory-motor experiences. Besides, the use of fingers in calculations is still very frequent at the beginning of formal schooling. Children who may be more proficient in finger-numeral configuration naming may already have a deeper understanding of number concepts and thus be more proficient in arithmetic. Also, in line with Fayol et al. (1998) and Badets et al. (2010) finger-numeral representations have an impact on arithmetic and even simple arithmetic operations are unconsciously supported by finger-numeral representation in adults.

Although reading and arithmetic involve multiple cognitive processes, common, and specific ones, it is interesting that number-specific RAN (digit and finger-numeral configuration RAN) predominantly predicted arithmetic and alphanumeric RAN primarily predicts reading.

Thus, the present study underscores the importance of assessing alphanumeric RANs when administering early readers’ screening batteries in order to identify and help children at risk at an early stage for developing later reading difficulties.

Although RANs explained a larger proportion of variance in reading than in arithmetic, we emphasize the importance of assessing children rapid naming of number-specific stimuli, such as finger-numeral configurations in early numeracy screening batteries. We investigated RAN only in typically developing children and future research on atypical arithmetic development is needed to investigate whether number-specific RANs (i.e., dice and finger-numeral configuration RAN) represent sound markers of later arithmetic difficulties.

To conclude, we propose that RAN acts like a multi-componential precursor skill for both reading and arithmetic skills (cf. Heikkilä et al., 2009). Administering rapid naming tasks at the beginning of first grade would give educational practitioners a valuable hint on children’s developing reading and arithmetic skills and may foster individualized instruction and intervention programs to support children at risk for later learning difficulties.

### Limitations and General Conclusion

One shortcoming of the present study is that our findings may only generalize to typically developing children, who do not present poor reading and/or poor arithmetic skills, and to the developmental stage of the participants (i.e., beginning of formal schooling). Moreover, our results may only generalize to orthographies with comparable features as French. A further limitation is that we did not control for other prominent cognitive abilities related to reading and arithmetic such as for instance general cognitive abilities, phonological awareness, and working memory. The focus of the present research was to examine the general and specific RAN contributions to reading and arithmetic and we do not claim that other cognitive skills are less predictive and important than RAN. Furthermore, we did not investigate the relationship between RAN and spelling skills. In a recent longitudinal study, RAN was strongly related to reading skills and a little less to spelling when compared with phonological awareness (Furnes and Samuelsson, 2011). Therefore, it would have been interesting to investigate the general and specific contributions of RAN also to spelling skills at the beginning of first grade.

In summary, the present research examined the structure of RAN in first graders and showed that different RAN performances represent a unique factor when adopting a general cognitive perspective. However, from a clinical perspective, when using RAN as a screener of young children’s future reading and arithmetic skills, it is important to look at specific contributions of RAN tasks and select the task which is most predictive of children’s reading and arithmetic progress. Indeed, our results emphasize the use of alphanumeric RAN tasks, specifically the vowel naming tasks, when predicting reading accuracy and digit RAN when predicting reading speed. Arithmetic fluency has been uniquely and primarily predicted by finger-numeral configuration RAN. Possibly, number-specific RAN tasks may be helpful in clinical contexts when screening children with low arithmetic skills. Future research should therefore investigate this hypothesis. Overall, our results highlight the inclusion of RAN tasks in early cognitive screening batteries, critical for early identification of later learning difficulties.

## Author Contributions

CH, RM, and MF discussed the current literature, developed the research questions and hypotheses. CH, RM, and MF designed the study design and the tests. MF collected the data. CH and RM analyzed the data. CH wrote the manuscript and finalized the tables and figures. MF reviewed and edited the manuscript.

## Conflict of Interest Statement

The authors declare that the research was conducted in the absence of any commercial or financial relationships that could be construed as a potential conflict of interest.

## Supplementary Material

**APPENDIX 1 |** Finger-numeral configuration RAN task.
