# Supplementary figures and images for: General and Specific Contributions of RAN to Reading and Arithmetic Fluency in First Graders: A Longitudinal Latent Variable Approach
Source: Front Psychol. 2017 Oct 6;8:1746. doi: 10.3389/fpsyg.2017.01746 (PMC5635811; doi:10.3389/fpsyg.2017.01746)

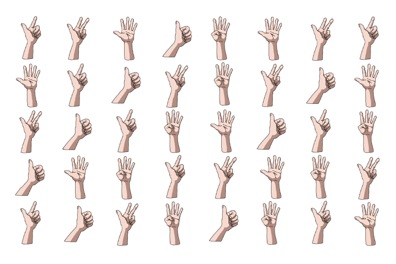

Supplement: Supplementary file 1 [file Appendix_1.JPEG]
