# Supplementary material for: General and Specific Contributions of RAN to Reading and Arithmetic Fluency in First Graders: A Longitudinal Latent Variable Approach
Source: Front Psychol. 2017 Oct 6;8:1746. doi: 10.3389/fpsyg.2017.01746 (PMC5635811; doi:10.3389/fpsyg.2017.01746)
Supplement: Supplementary file 2 [file Appendix_2.DOCX]

**Appendix 2**

Table 4. VIF indexes for the inter-correlated RAN measures in first graders (N= 122)

|  | **VIF indexes among independent RAN measures** | | | | | | |
| --- | --- | --- | --- | --- | --- | --- | --- |
|  | **Color RAN** | **Object RAN** | **Vowel RAN** | **Consonant RAN** | **Digit RAN** | **Finger RAN** | **Dice RAN** |
| **Color RAN** | - | 2.04 | 2.82 | 1.64 | 2.85 | 2.30 | 3.27 |
| **Object RAN** | 2.36 | - | 2.91 | 1.65 | 2.84 | 2.30 | 3.19 |
| **Vowel RAN** | 2.38 | 2.13 | - | 1.58 | 2.86 | 2.14 | 3.17 |
| **Consonant RAN** | 2.43 | 2.11 | 2.76 | - | 2.83 | 2.30 | 3.42 |
| **Digit RAN** | 2.42 | 2.09 | 2.88 | 1.63 | - | 2.12 | 3.19 |
| **Finger RAN** | 2.45 | 2.12 | 2.70 | 1.66 | 2.65 | - | 3.37 |
| **Dice RAN** | 2.34 | 1.98 | 2.70 | 1.66 | 2.69 | 2.27 | - |
